# Expressions of consent and pleasure: the development and initial validation of sexual video clips specifically created for sex research

**DOI:** 10.1038/s41598-025-22527-9

**Published:** 2025-11-04

**Authors:** Mariana L. Carrito, Pedro Nobre, Erick Janssen

**Affiliations:** 1 Centre for Psychology at University of Porto (CPUP), University of Porto, Porto, Portugal; 2 Institute for Family and Sexuality Studies, Department of Neurosciences, KU Leuven, Leuven, Belgium; 3 Faculdade de Psicologia e de Ciências da Educação, Uniersidade do Porto, Rua Alfredo Allen, Porto, 4200–135 Portugal

**Keywords:** Sexually explicit videos, Sex research, Sexual arousal, Affective responses, Sexual aggression, Consent, Human behaviour, Emotion, Physiology

## Abstract

Sexually explicit videos are widely used in sexuality research to induce sexual arousal, yet there remains a critical need for validated and standardized audiovisual materials specifically designed for research purposes. This study validated a newly developed series of sexual video clips for use in psychophysiological sex research. Fifty heterosexual men viewed three sexual video clips depicting sexual interactions between a male and female actor and representing different levels of engagement and consent. Participants rated their sexual arousal, emotional responses, and perceptions of the female actor’s arousal and consent. Each video effectively conveyed engagement, ambiguity, or distress and elicited distinct emotional and perceptual responses. The engagement video elicited the highest levels of sexual arousal, pleasure, and positive emotions, while the distress video evoked the strongest negative affect and general arousal. The ambiguity video elicited responses similar to those for the distress video. Individual differences in sexual excitation and inhibition influenced emotional and sexual responses, and sexual aggression perpetration was linked to reduced sensitivity to lower-consent cues in the ambiguity video. These findings demonstrate the value of these novel sexual film clips in enhancing the precision and validity of sex research, especially in the study of sexual aggression.

**Supplementary Information:**

The online version contains supplementary material available at 10.1038/s41598-025-22527-9.

## Introduction

Sexual arousal is a complex and multifaceted construct that has relevance to our understanding of a wide range of phenomena, including sexual (dys)function, sexual orientation, sexual risk-taking, and sexual aggression. To conduct meaningful research in this area, sex researchers have invested in employing innovative experimental paradigms that allow for the systematic and standardized assessment of sexual arousal and its underlying processes. Such paradigms include the use of sexually explicit videos as experimental stimuli, which provide researchers with a standardized way to examine individuals’ responses and behaviors related to sexual content, and their use has been particularly instrumental in exploring various aspects of human sexual arousal^1^.

Previous studies have repeatedly demonstrated the effectiveness of sexually explicit films in inducing sexual arousal. In fact, there is compelling evidence that films generally generate higher response levels than other sexual stimuli like stories, still images, or fantasy instructions^1–4^. Moreover, explicit sexual stimuli result in higher levels of sexual arousal than stimuli with more romantic and less explicit content e.g^5^. Because of their generally high effectiveness in inducing sexual arousal in a laboratory context, sexually explicit films have been used to investigate the role of cognitive and affective processes in sexual arousal e.g^6–9^, gender differences in sexual arousal^10–12^, links between sexual arousal and sexual orientation e.g^13^, hypersexuality e.g^14^, sexual risk-taking e.g^15^, and sexual aggression e.g^16^.

To leverage the effectiveness of films, sex researchers commonly use commercially available erotic films due to their ease of duplication, editing, and the convenience of standardization^17^. However, relying on publicly available sexually explicit materials limits researchers’ control over stimulus content and the associated intended affective reactions and sexual response levels. Mixed and contradictory findings on responsiveness to explicit sexual content may, in part, be attributed to uncontrolled variations, even within a specific category (e.g., heterosexual sexual interactions), impacting affective and sexual responses^18^. For instance, men and women in video clips used in a study may vary in physical characteristics and perceived attractiveness. Also, it is not uncommon for researchers to use video clips within a single study that vary in context (e.g., indoor/outdoor, conversation) and atmosphere (e.g., lighting).

Moreover, researchers and ethics committees are increasingly focused on ensuring that sexually explicit content used in research studies has been created without coercion or exploitation. Ideally, researchers only use stimulus sets known to have been produced with the full consent of the individuals involved and under ethically and legally acceptable circumstances. Commercially available materials can pose challenges in meeting these requirements. The need for ethically created and obtained stimuli is even more critical when the content is intended to depict nonconsensual sexual interactions, as is the case, for example, in psychophysiological studies of sexual aggression.

A growing number of studies have shown that individuals convicted of sexual offenses, as well as sexually coercive men recruited from community samples, may exhibit higher levels of sexual arousal in response to sexual stimuli, including coercive ones, compared to noncoercive men^19,20^. In addition, men with no history of sexual aggression may also show some level of sexual arousal to sexual stimuli that include coercive behavior^21^. While there is ongoing debate about the extent to which sexual versus nonsexual motives, such as power, contribute to sexual aggression, research strongly suggests that showing signs of sexual arousal to coercive sexual stimuli is predictive of a man’s preference for non-consensual sex^22^. In addition to sexual response patterns, researchers studying sexual aggression often include measures of emotional and affective responses towards depictions of sexual violence, including the experience of fear, disgust, anger, or empathy. These emotional responses have been found to play a role in shaping individuals’ perceptions and histories of sexual violence, as well as influencing their willingness to intervene or support potential victims^23^. For example, research has shown that sexually aggressive men may exhibit heightened levels of negative affect, such as anger, anxiety, and hostility^20,24^. Also, Peterson and Janssen^25^, in a sample of college students, found that the presence of both positive and negative affect, or ambivalent affect, predicted subjective sexual arousal to explicit sexual films, including ones depicting sexual aggression.

## Current study

The use and validation of ethically created and obtained sexually explicit video sets are crucial for safeguarding research integrity and ensuring the reliability and validity of experimental findings. The goal of the current study is to introduce and provide the initial validation of a new set of sexual video clips specifically created for sex research. The videos of this set were produced by a video production company that specializes in erotic short films, which is dedicated to promoting ethical porn and is committed to following legal guidelines for the production and distribution of adult content. The videos were created to be available with restricted access to authorized researchers across various fields, including sex research and aggression studies.

In this study, heterosexual men’s sexual and emotional responses to the newly created video clips were evaluated. In total, three videos were utilized, all depicting sexual interactions between the same male and female actor, but differing in the degree of expressed engagement and consent. The scenarios ranged from consensual interactions, to ambiguous situations, and to explicit portrayals of distress and non-consent. Responses encompassed self-reported levels of general arousal, sexual arousal and desire, and positive and negative affect. To examine whether the videos effectively conveyed the intended variations in consent and pleasure, we also asked participants to rate the female actor’s levels of sexual arousal, pleasure, consent, willingness to engage in sex (cf. wantedness)^26^, as well as her emotional reactions. The study design, hypotheses, and data analysis plan were preregistered on the Open Science Framework (OSF; https://osf.io), and this paper focuses on a subset of the planned analyses, with the remaining analyses to be reported in future work. The preregistration can be accessed at https://osf.io/386gz.

## Materials and methods

### Participants

Participants were recruited through university advertisements, student email lists, and social media. Men were eligible for participation if they were between 18 and 40 years old, self-identified as heterosexual, and were fluent in Portuguese. According to an a priori power analysis using G*Power software with 0.95 power at a 0.05 alpha error probability, we would need at least 42 participants to detect a medium effect (partial eta square = 0.06) in a repeated-measures ANOVA with three video conditions. A total of 50 male volunteers, with a mean age of 24.34 (SD = 4.92, range = 18–38) years, participated in the experiment. Overall, participants were vaguely familiar with Consensual Non-Consent (CNC) Kink practices (Med = 2, IQR = 4), found the actress in the videos to be mildly attractive (Med = 3; IQR = 2), and felt lightly immersed in the action during the experiment (Med = 2, IQR = 2).

### Stimuli

Participants were presented with three videos that conveyed sexually explicit interactions from a first-person perspective (point-of-view, or POV). The videos, with a duration of 90 s each, showed a female actor engaging in sexual intercourse with an offscreen male actor. The three videos differed only in the facial and behavioral expressions of the female actor, which depict, respectively, consent and engagement, ambiguity, and non-consent and distress:

- Consent and engagement: This video clip emphasizes the female actor’s experience of pleasure. She smiles, looks sensual, bites her lip, makes eye contact (looks into the camera), makes sounds indicative of pleasure and bliss (light sighs, giggles, moans, groans), breathing intensely. She also touches herself, caressing her own body.
- Ambiguity: In this video clip, the female actor has a neutral facial expression, with her mouth closed and without making any sound, while maintaining eye contact. She does not show signs of pleasure, but she also does not show signs of distress or avoidance.
- Non-consent and distress: In this video clip, the male actor forces the female actor to have intercourse with him. The female actor shows clear signs that she does not want to engage in sexual activity with him, pushing him away. She is not receiving any pleasure from the interaction and expresses distress and other negative emotions.

Given the sensitive material of this video set, it will be available only for research purposes and upon request. Researchers interested in accessing the materials must meet ethical and institutional requirements outlined in the Materials Access Statement [https://osf.io/byfh2/?view_only=f8e30d87bf8643c8846a633a973b3cd0].

### Procedure

After written informed consent was obtained, participants were seated in front of a computer screen in a dimly lit and sound-attenuated room. Participants were presented with three sexually explicit videos and responded to several questions enquiring about their perceptions of the contents and the elicited emotions. Each explicit video was preceded by a 90-second neutral video clip to facilitate a return to baseline response before its visualization. The order of sexually explicit clips was counterbalanced between participants.

At the start of the experiment and following each sexually explicit clip, participants were asked to rate on a 100-point Visual Analogue Scale (VAS) their general arousal (i.e., perceived state of heightened physiological and psychological activity or alertness), sexual arousal (i.e., subjective experience of sexual activation associated with sexual excitement), and desire (i.e., thoughts, fantasies and feelings of sexual interest). They were also asked to complete the Positive and Negative Affect Scale^27,28^ (1–5 Likert scale). Exclusively following sexually explicit video clips, participants also indicated, using a 100-point VAS, an assessment of sexual arousal, pleasure (i.e., satisfaction and enjoyment derived from erotic experience), consent (i.e., agreement to engage in sexual activity), and wantedness (i.e., willingness to engage in sex) as experienced by the female actor. The order of presentation of questions within each arousal/emotional category was randomized.

At the end of the experiment, participants rated, using a seven-point Likert scale, the attractiveness of the female actor and level of experienced immersion (the degree to which a viewer feels immersed in a film clip), and their familiarity with CNC Kink practices. Also, at the end of the session, participants completed a short demographic questionnaire inquiring about age and nationality and confirming gender and sexual orientation. Lastly, they were asked to complete the Sexual Experiences Survey/Perpetration Version (SES-P)^29^ and the Sexual Inhibition (SIS) and Sexual Excitation (SES) Scales^30,31^. Regarding the SES-P scale, men were later divided into two groups (perpetrators, *N* = 12, and non-perpetrators, *N* = 38) based on whether they indicated having engaged in any kind of sexual aggression perpetration since the age of 14.

All participants received a 10€ value voucher. The study received ethical approval from the Ethics Committee of the Faculty of Psychology and Education Sciences—University of Porto. All aspects of the study, including stimulus content and dataset’s availability, were assessed and approved by the ethics committee and conducted in accordance with the 1964 Helsinki Declaration and its later amendments.

### Data analyses

The data were analyzed using SPSS v29. Descriptive statistics for the three videos (standard deviations and confidence intervals (CI) for each stimulus in each evaluative dimension) can be found as Supplementary Table S1 online. For the ratings on self-reported arousal (including measures of “desire”, “general arousal”, and “sexual arousal”), a repeated-measures MANOVA was conducted with type of videos (consent and engagement, ambiguity, non-consent and distress) as within-subjects factor, to examine the effects of the different video conditions. The same approach was applied when exploring perceived actress’ arousal (comprising “sexual arousal”, “pleasure”, “consent”, and “wantedness”). All MANOVAs were followed by univariate analyses when significant effects were found. Repeated Measures ANOVAs were also conducted to explore affective responses (measured through PANAS). Greenhouse-Geisser corrections were applied for sphericity violations and corrected degrees of freedom were reported in such cases. Statistical significance was set at *p* <.05. For every significant effect, pairwise comparisons were examined, incorporating Bonferroni corrections. Spearman correlations were computed to explore associations between the participants’ Sexual Inhibition/Sexual Excitation Scales indices (SIS/SES) and the scores of their own/actress arousal ratings. Mann–Whitney U tests were conducted for comparing groups based on sexual aggression perpetration (SES-P), along with rank-biserial correlations as effect sizes.

## Results

### Construct validity of videos

#### Actress’ perceived responses

A 3-way repeated-measures MANOVA was conducted to examine the effects of video (engagement, ambiguity, distress) on four dependent variables relevant to how the actress was perceived (actress’ sexual arousal, pleasure, consent, and wantedness). This analysis revealed a significant main effect of video (F (8, 190) = 81.25, *p* <.001, η_p_^2^ = 0.774). Univariate follow-up analyses revealed a significant effect of video on all four variables: perceived actress’ sexual arousal (F (1.65, 81.06) = 369.12, *p* <.001, η_p_^2^ = 0.883), pleasure (F (1.74, 85.22) = 437.80, *p* <.001, η_p_^2^ = 0.899), consent (F (1.76, 86.04) = 235.04, *p* <.001, η_p_^2^ = 0.827), and wantedness (F (2, 98) = 432.88, *p* <.001, η_p_^2^ = 0.898).

**Fig. 1:**
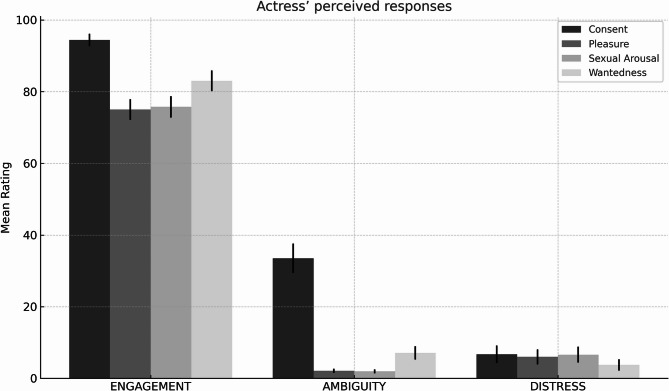
Mean ratings of the actress’s perceived consent, pleasure, sexual arousal, and wantedness across the *Engagement*, *Ambiguity*, and *Distress* conditions. Error bars represent standard error of the mean (SEM). Ratings were provided on a 0–100 scale.

Participants considered the actress to have experienced the highest sexual arousal (M = 75.76, SE = 2.99), pleasure (M = 75.00, SE = 2.90) and wantedness (M = 83.03, SE = 2.88) in the engagement video compared to the other videos (see Fig. 1). There was no difference between sexual arousal responses attributed to the ambiguity (Sexual Arousal: M = 1.97, SE = 0.53; Pleasure: M = 2.12, SE = 0.57; Wantedness: M = 7.12, SE = 1.85) and distress (Sexual Arousal: M = 6.59, SE = 2.21; Pleasure: M = 6.00, SE = 2.07; Wantedness: M = 3.76, SE = 1.59) videos.

Perceived consent was the only variable that was statistically different between the three videos. Participants considered the actress to have experienced the highest level of consent in the engagement video (M = 94.41, SE = 1.74), followed by the ambiguity video (M = 33.50, SE = 4.10), which in turn was associated with a higher level of consent comparing to the distress video (M = 6.73, SE = 2.44).

#### Participants’ arousal and sexual responses

A 3-way repeated-measures MANOVA was conducted, examining the effects of video (engagement vs. ambiguity vs. distress) on the dependent variables (general arousal, sexual arousal and desire). This analysis revealed a significant main effect of video (F (6, 192) = 38.73, *p* <.001, η_p_^2^ = 0.548).

**Fig. 2:**
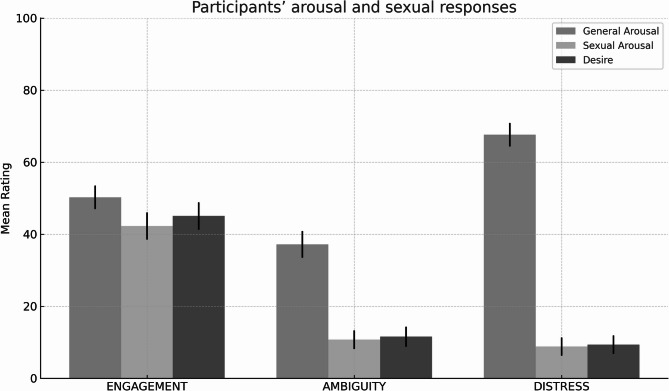
Mean ratings of participants’ general arousal, sexual arousal, and sexual desire across the *Engagement*, *Ambiguity*, and *Distress* conditions. Error bars indicate standard error of the mean (SEM). Ratings were provided on a 0–100 scale.

Subsequent univariate analyses revealed a significant effect of video specifically on general arousal (F (2, 98) = 27.77, *p* <.001, η_p_^2^ = 0.362). General arousal was higher in the distress video (M = 67.62, SE = 3.25) than in other videos (see Fig. 2). Conversely, during the engagement video, participants reported an average level of general arousal (M = 50.26, SE = 3.24), which was higher than the level reported during the ambiguity video (M = 37.19, SE = 3.70).

A significant effect was also verified for sexual arousal, F (1.67, 81.70) = 69.26, *p* <.001, η_p_^2^ = 0.586, and desire, F (1.45, 71.08) = 87.11, *p* <.001, η_p_^2^= 0.640. During the engagement video, participants reported higher sexual arousal (M = 42.29, SE = 3.79) and desire (M = 45.07, SE = 3.78) compared to the other videos, even though the scores were relatively low. Both the ambiguity (Sexual Arousal: M = 10.74, SE = 2.60; Desire: M = 11.56, SE = 2.75) and the distress videos (Sexual Arousal: M = 8.82, SE = 2.51; Desire: M = 9.36, SE = 2.53) resulted in low sexual arousal/desire among participants.

#### Participants’ affect

**Fig. 3:**
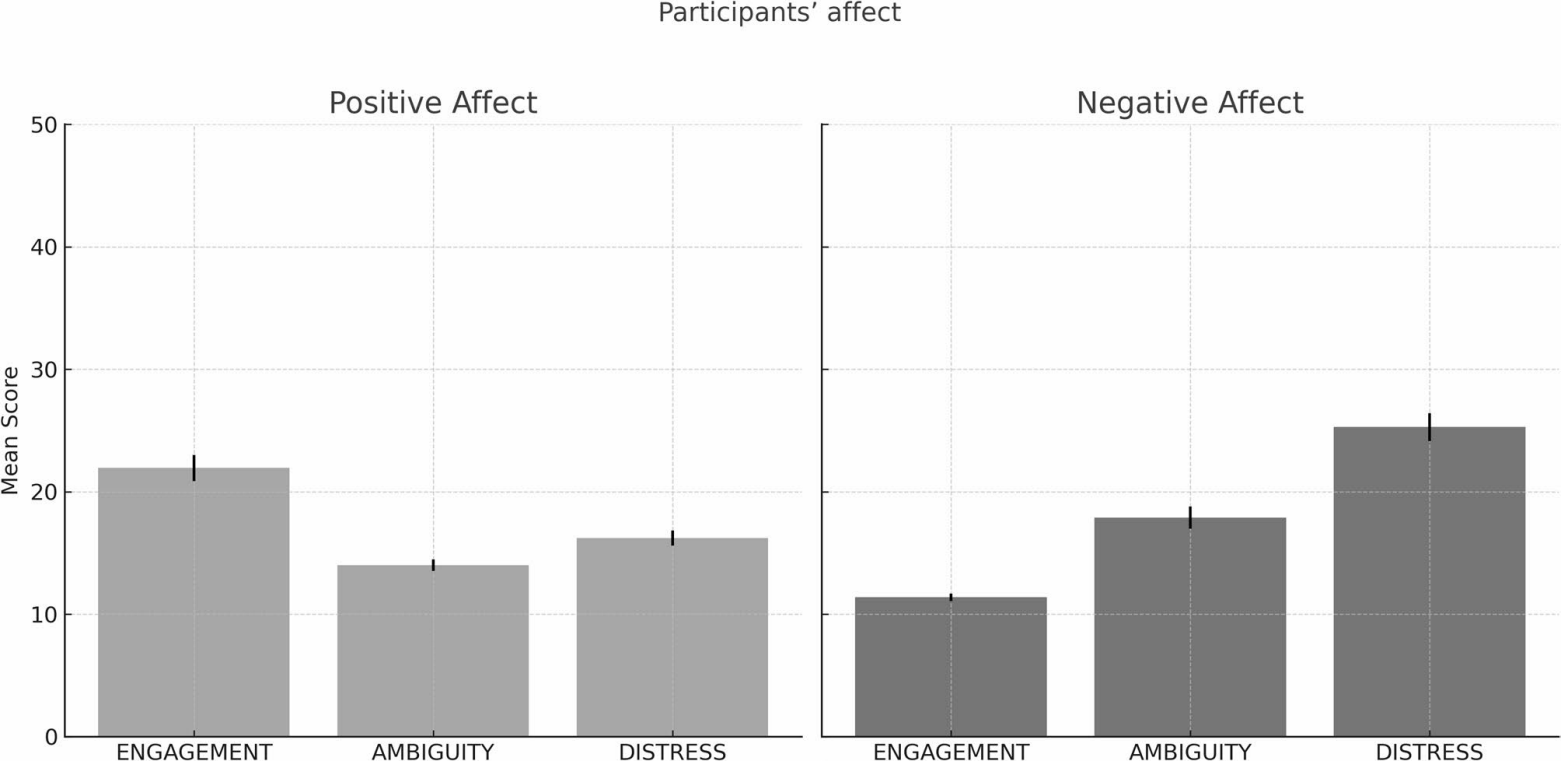
Participants’ positive and negative affect across the *Engagement*, *Ambiguity*, and *Distress* conditions, measured using the PANAS scale (range: 0–50). Error bars represent standard error of the mean (SEM).

We found a significant effect of video when performing a repeated-measures ANOVA for the positive affect score measured through PANAS, F (1.36, 66.86) = 52.40, *p* <.001, η_p_^2^ = 0.517. Participants’ affect was mildly positive in the engagement video (M = 21.94, SE = 1.08), lower in the distress video (M = 16.22, SE = 0.61), but even significantly lower in the ambiguity video (M = 14.00, SE = 0.48) (see Fig. 3).

There was also a significant effect of video when performing a repeated-measures ANOVA for the negative affect score, F (2, 98) = 97.46, *p* <.001, η_p_^2^ = 0.665. Participants’ affect was mildly negative in the distress video (M = 25.28, SE = 1.15), lower in the ambiguity video (M = 17.90, SE = 0.91), with the lowest average score found for the engagement video (M = 11.38, SE = 0.31).

### Association with sexual inhibition/excitation and sexual aggression

Correlational analyses were performed to explore whether the propensity for sexual inhibition or excitation was related to participants’ perceptions and affective responses. Spearman correlation analyses showed that participants’ propensity to sexual excitation (SES) correlated negatively with perceived actress consent in the engagement video (*r*_*sp*_ = − 0.294, *p* =.038). SES also significantly correlated with sexual desire (*r*_*sp*_ = 0.457, *p* <.001) and sexual arousal (*r*_*sp*_ = 0.438, *p* =.001) felt during the engagement condition, indicating that participants with higher sexual excitation propensity reported higher sexual responsivity during the engagement video. Sexual inhibition due to the threat of performance failure (SIS1) correlated positively with the negative affect felt during the engagement video (*r*_*sp*_ = 0.402, *p* =.004). Sexual inhibition due to the threat of performance consequences (SIS2) negatively correlated with general arousal reported during the engagement video (*r*_*sp*_ = − 0.301, *p* =.034) and positively correlated with the negative affect felt during the ambiguity video (*r*_*sp*_ = 0.344, *p* =.014).

Mann–Whitney U tests were conducted to examine differences in emotional and sexual responses between men who reported sexual aggression perpetration (*n* = 12) and those who did not (*n* = 38). Significant group differences were observed for general arousal in the ambiguity condition (U = 113.5, *p* =.009, rank-biserial correlation = 0.502), with non-aggressors reporting higher general arousal than aggressors.

A significant difference was also found for negative affect in response to the ambiguity video condition (U = 141.0, *p* =.047, rank-biserial correlation = 0.383), with non-aggressors reporting higher negative affect than aggressors. No other significant differences emerged between groups across the remaining measures (all *p*_*s*_ > 0.05).

## Discussion

This study presented and validated a new set of three videos created specifically for use in sex research. By exploring their impact on participants’ sexual and emotional subjective responses, we aimed to confirm whether the three videos portrayed, as intended, expressions of engagement, ambiguity, and distress, respectively. The results demonstrated a clear and significant impact of video on all scores, indicating that the content is perceived to convey varied interactions and elicit significantly different reactions in participants. The three videos effectively communicated different aspects of sexual interactions to viewers as participants identified one video portraying a consensual situation, another describing a non-consensual interaction, and the third depicting an unclear level of consent.

Particularly regarding the engagement video, collected data confirms that this clip is perceived as depicting a genuine engagement. Participants rated this video as showing a higher level of sexual arousal and pleasure from the actress, indicating her clear interest in pursuing such interaction. Additionally, participants in this condition reported higher levels of sexual arousal, desire, positive emotional state, and affect when describing their own levels of arousal. These results confirm that the engagement video is capable of eliciting sexual arousal, similar to other sexual audiovisual stimuli used in previous laboratory studies^32^.

Conversely, the distress condition was also proven effective by being perceived as the one where the actress displays clear signs of lack of sexual arousal, pleasure, and “wantedness”. This video evokes lower sexual arousal and desire in participants while activating their general arousal. Such heightened general arousal may be related to the negative emotions reported by participants while watching this content, as this condition elicited the highest level of negative affect scores. In fact, previous research has shown audiovisual rape scenes to elicit both higher negative affect and physiological arousal (indexed by increased facial electromyography), compared to stimuli depicting nonviolent male-female interactions^33^.

Interestingly, despite the varying levels of consent in the three videos, many perceptions and reactions were similar between the distress and ambiguity videos. Participants believed that the actress experienced similar sexual arousal, pleasure, and “wantedness” in both scenarios. The ambiguity and distress stimuli resulted in low sexual arousal and desire among participants. This similarity in sexual arousal responses could be linked to the negative emotions experienced during the ambiguity video.

Regarding potential influences of individual differences, it is noteworthy that a positive correlation was found between individuals’ propensity for excitation and desire/sexual arousal felt during the engagement video. This suggests that individuals who are naturally more prone to excitement and desire are more likely to experience heightened sexual arousal when exposed to content depicting genuine engagement. Moreover, we found that individuals who had perpetrated acts of sexual aggression since the age of 14 reported lower levels of negative affect and general arousal while viewing the ambiguity video. Since consent cues are not entirely clear in the ambiguity video, this effect might suggest a lack of empathic skills among participants who fail to reject ambiguous consent scenarios in the same manner as non-perpetrators^34^. This relationship warrants further investigation in future studies, as it could provide valuable insights for applications aimed at preventing sexual aggression.

### Potential application of validated videos

Researchers in the fields of psychology and sex research can utilize validated videos as standardized stimuli in studies related to sexual behavior, emotional responses, and social perception. The videos can provide a consistent and controlled method for eliciting specific emotional and physiological responses in participants, thereby enhancing the reliability and comparability of research findings. The preoccupation with using validated laboratory paradigms has been addressed before, namely regarding the study of sexual violence^22^, but many of the stimuli used still rely on publicly available material^35^. The videos presented here provide a unique opportunity to rigorously isolate cues related to social interaction that may occur during a sexual relationship. Variable isolation is crucial to ensuring proper experimental control and obtaining accurate results. This involves carefully managing and manipulating individual variables to minimize potential confounding factors.

Using these materials should be approached with ethical considerations. Researchers and practitioners should be mindful of the potential impact of the stimuli on participants and take measures to minimize any potential adverse effects.

Consideration should be given to the potential implications of the research on individuals and communities, and efforts should be made to ensure that the application of the findings aligns with ethical standards and promotes positive societal outcomes.

### Limitations

One potential limitation that we can anticipate with these materials is their specificity in terms of shooting type and environment. We have prioritized experimental control by requesting producers to create a simple, minimalistic setting for all films and during all shooting. This approach aimed to minimize the influence of external visual and audio elements on our participants’ responses, thereby enhancing the validity of our findings. However, we acknowledge that this choice may limit engagement or visual appeal for some viewers, especially those not fans of POV movies. Less visual interest could affect arousal and emotional reaction, making it interesting to explore further through qualitative measurements or psychophysiological assessments integrating genital measurements. Also, while a MANOVA framework was used to explore within-subject differences across conditions in this study, we acknowledge the limitations of this method, particularly with smaller samples. Future research should consider applying more flexible multivariate approaches, such as linear mixed-effects models, to better account for inter-individual variability and repeated measures’ structure.

## Conclusion

This study provides a set of three validated videos depicting diverse sexual interactions, representing the presence and absence of pleasure and consent. Findings confirm that the presented stimuli effectively elicit corresponding emotional and sexual responses from participants. Each video successfully conveyed its intended narrative, with the engagement video depicting and evoking genuine arousal and positive emotional states, the distressing video provoking heightened general arousal alongside negative affect, and the ambiguity video generating nuanced consent, although being perceived as more prominently negative.

The videos developed in this study hold particular promise for advancing research into sexual aggression. By isolating specific cues related to consent, engagement, and distress, these stimuli provide researchers with a controlled framework for examining the psychological and emotional reactions that may be associated with aggressive behaviors and exploring the factors influencing perceptions of consent and coercion. Such investigations are critical for understanding the cognitive and emotional mechanisms underlying sexual aggression and for identifying risk factors that contribute to aggressive behaviors. Future studies should also consider integrating qualitative and psychophysiological measures to enrich understanding and address these challenges.

In summary, these validated stimuli represent a significant contribution to the toolkit of researchers investigating sexual behavior and emotional responses, offering standardized and ethically designed materials to foster reliable and meaningful insights in this sensitive field.

## Supplementary Information

Below is the link to the electronic supplementary material.

Supplementary Material 1

## Data Availability

The datasets analyzed during the current study are available in the OSF repository, [https://osf.io/byfh2/?view_only=f8e30d87bf8643c8846a633a973b3cd0].
